# Pterostilbene Suppresses both Cancer Cells and Cancer Stem-Like Cells in Cervical Cancer with Superior Bioavailability to Resveratrol

**DOI:** 10.3390/molecules25010228

**Published:** 2020-01-06

**Authors:** Hee Jeong Shin, Jang Mi Han, Ye Seul Choi, Hye Jin Jung

**Affiliations:** 1 Department of Life Science and Biochemical Engineering, Sun Moon University, Asan 31460, Korea; gmlwjd903@naver.com (H.J.S.); gkswkdal200@naver.com (J.M.H.); yesll96@naver.com (Y.S.C.); 2 Genome-based BioIT Convergence Institute, Asan 31460, Korea; 3 Department of Pharmaceutical Engineering and Biotechnology, Sun Moon University, Asan 31460, Korea

**Keywords:** cancer stem cell, cervical cancer, pterostilbene, resveratrol

## Abstract

Increasing studies have reported that cancer stem cells (CSCs) play critical roles in therapeutic resistance, recurrence, and metastasis of tumors, including cervical cancer. Pterostilbene, a dimethylated derivative of resveratrol, is a plant polyphenol compound with potential chemopreventive activity. However, the therapeutic effect of pterostilbene against cervical CSCs remains unclear. In this study, we compared the anticancer effects of resveratrol and pterostilbene using both HeLa cervical cancer adherent and stem-like cells. Pterostilbene more effectively inhibited the growth and clonogenic survival, as well as metastatic ability of HeLa adherent cells than those of resveratrol. Moreover, the superior inhibitory effects of pterostilbene compared to resveratrol were associated with the enhanced activation of multiple mechanisms, including cell cycle arrest at S and G2/M phases, induction of ROS-mediated caspase-dependent apoptosis, and inhibition of matrix metalloproteinase (MMP)-2/-9 expression. Notably, pterostilbene exhibited a greater inhibitory effect on the tumorsphere-forming and migration abilities of HeLa cancer stem-like cells compared to resveratrol. This greater effect was achieved through more potent inhibition of the expression levels of stemness markers, such as CD133, Oct4, Sox2, and Nanog, as well as signal transducer and activator of transcription 3 signaling. These results suggest that pterostilbene might be a potential anticancer agent targeting both cancer cells and cancer stem-like cells of cervical cancer via the superior bioavailability to resveratrol.

## 1. Introduction

Cervical cancer is one of the most common types of female malignant tumor, with worldwide incidence of more than 500,000 cases and mortality rate of 9% per year [1]. High-risk human papillomavirus (HPV) types such as HPV-16 and -18 are known to cause cervical cancer through the overexpression of viral oncoproteins E6 and E7 [2]. Although the worldwide death rate from cervical cancer has declined due to the current treatment modalities, including HPV vaccines, surgery, radiation therapy, and chemotherapy, the cancer recurrence, metastasis, and the adverse drug effects remain major problems [3]. Therefore, safer and more effective therapeutic options are needed to improve the treatment of cervical cancer.

Accumulating evidence has demonstrated that cancer stem cells (CSCs), a small subpopulation of tumor cells with self-renewal and multi-lineage differentiation capacities, crucially drive the development, metastasis, relapse, and chemo/radio-resistance of cervical cancer [4,5]. In addition, HPV oncoprotein E6 has been found to be involved in self-renewal and maintenance of stemness in cervical CSCs by upregulating Hes1, a downstream gene of Notch1 [6]. HPV16 E7 also upregulates the expression of stemness-related genes such as Oct3/4, Sox2, Nanog, and fibroblast growth factor 4 to maintain the self-renewal capacity of cervical CSCs [7]. Accordingly, targeting the cervical CSCs is a promising therapeutic strategy for the high-risk HPV-positive cervical cancer. 

**Figure 1:**
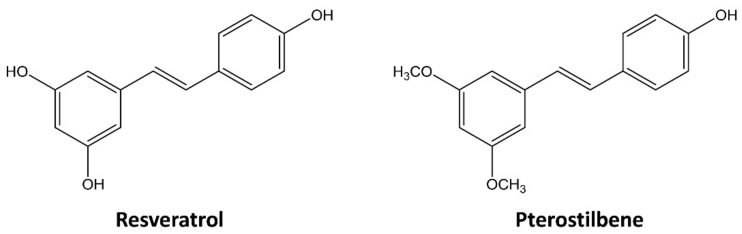
Chemical structures of resveratrol and pterostilbene.

Various scientific studies have suggested the potential of natural active compounds isolated from plants or herbs for prevention and treatment of cancer [8,9]. Stilbenes are a class of polyphenolic compounds and naturally found in various dietary sources, such as grapes, blueberries, red wine, peanuts, and some medicinal plants [10]. Recently, stilbenes such as resveratrol (3,4′,5-trihydroxy-trans-stilbene) and its dimethylated analog, pterostilbene (trans-3,5-dimethoxy-4′-hydroxystilbene), have received considerable attention due to their potent antioxidant, anti-inflammatory, antidiabetic, and anticarcinogenic properties (Figure 1) [11,12]. Resveratrol and pterostilbene have been considered as excellent anticancer agents because of their low toxicity and abilities to regulate multiple molecular signaling pathways involved in cancer progression [13,14]. However, resveratrol has a low bioavailability that may lower its biological efficacy, while pterostilbene is more lipophilic, and thus, it exhibits better bioavailability [15]. Pterostilbene shows stronger antiproliferative and apoptotic effects than those shown by resveratrol in the human colon and cervical cancer cells [16,17]. However, the therapeutic effect and anticancer mechanism of pterostilbene against cervical CSCs compared to resveratrol have not been studied.

Here, anticancer effects of resveratrol and pterostilbene were compared using both HeLa cervical cancer adherent and stem-like cells. The abilities of the two compounds to suppress growth, migration, and stemness of HeLa cells were evaluated and the underlying molecular mechanisms were further explored. The results revealed that pterostilbene more effectively inhibited the stem-like properties of HeLa cells than resveratrol through stronger downregulation of specific CSC markers and signal transducer and activator of transcription 3 (STAT3) signaling. This is the first study to demonstrate the potential inhibitory activity of pterostilbene against cervical cancer cell stemness.

## 2. Results

### 2.1. Pterostilbene Inhibited the Growth of Cervical Cancer Cells with Higher Potency Compared to Resveratrol

**Figure 2:**
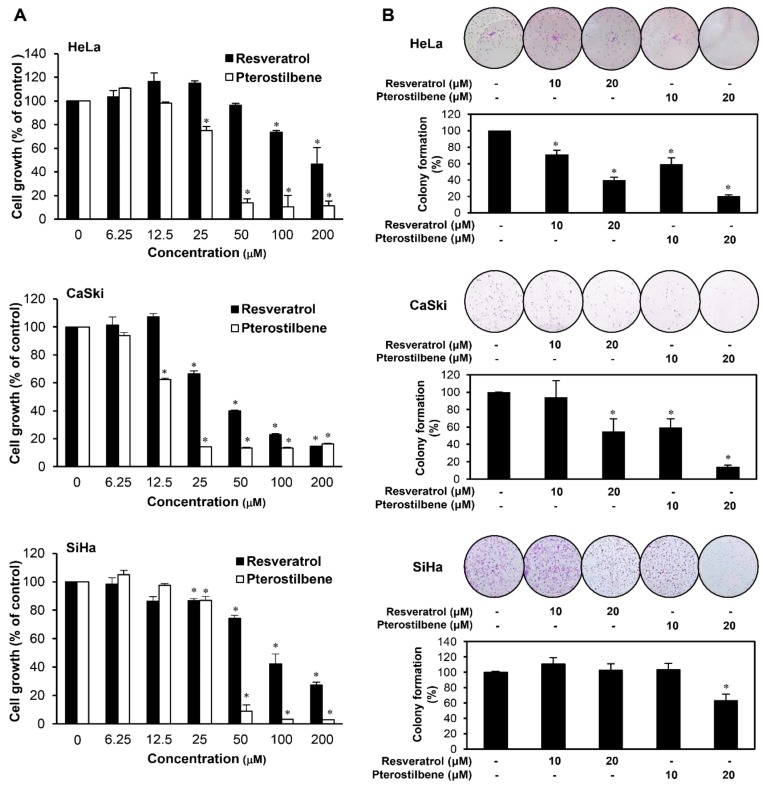
Growth inhibitory effects of resveratrol and pterostilbene on HeLa, CaSki, and SiHa cells. (**A**) The effects of resveratrol and pterostilbene on the growth of HeLa, CaSki, and SiHa adherent cells. The cells were treated with increasing concentrations of the two compounds (0–200 μM) for 72 h, and cell growth was measured by 3-(4,5-dimethylthiazol-2-yl)-2,5-diphenyltetrazolium bromide (MTT) assay. (**B**) The effects of resveratrol and pterostilbene on the colony forming ability of HeLa, CaSki, and SiHa adherent cells. The cells were incubated in the absence or presence of the two compounds (10 and 20 μM) for seven days. The cell colonies were detected by crystal violet staining. * *p* < 0.05 versus the control.

First, we compared the inhibitory effects of resveratrol and pterostilbene on the growth of HeLa, CaSki, and SiHa cervical cancer adherent cells using the 3-(4,5-dimethylthiazol-2-yl)-2,5-diphenyltetrazolium bromide (MTT) assay at various concentrations (0–200 μM). Resveratrol and pterostilbene suppressed the growth of HeLa, CaSki, and SiHa cells in a concentration-dependent manner (Figure 2A). The results showed that pterostilbene (IC_50_ = 32.67 μM for HeLa; 14.83 μM for CaSki; 34.17 μM for SiHa) exhibited stronger growth inhibitory effect than resveratrol (IC_50_ = 108.7 μM for HeLa; 44.45 μM for CaSki; 91.15 μM for SiHa). Next, we evaluated the effects of pterostilbene and resveratrol on the colony formation of HeLa, CaSki, and SiHa adherent cells. The colony forming ability of the cells was more effectively inhibited by pterostilbene than resveratrol (Figure 2B). These data demonstrate that pterostilbene is more potent in suppressing the growth and clonogenicity of cervical cancer adherent cells compared with resveratrol. 

### 2.2. Pterostilbene Exhibited Stronger Migration Inhibitory Effect than Resveratrol in Cervical Cancer Cells

**Figure 3:**
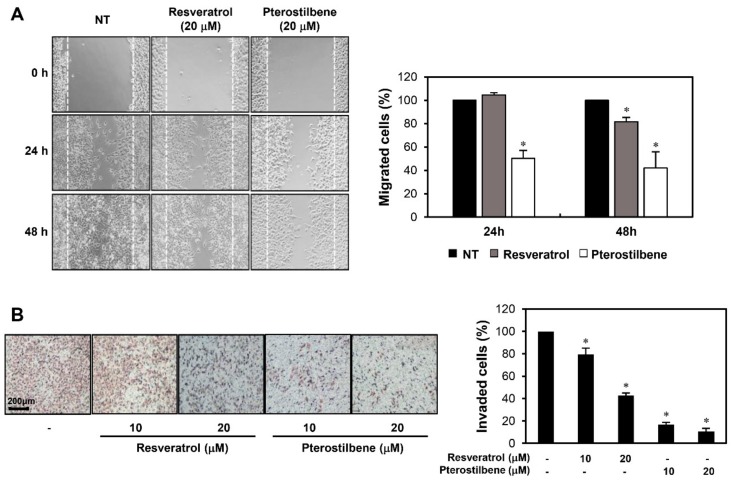
Effects of resveratrol and pterostilbene on the metastatic ability of HeLa cells. (**A**) The effects of resveratrol and pterostilbene on the migration of HeLa adherent cells. The migratory potential of HeLa cells was analyzed using a wound healing assay. The cells were incubated in the absence or presence of the two compounds (20 μM) for 48 h. The cells that migrated into the gap were counted using an optical microscope. Dotted white lines indicate the edge of the gap at 0 h. (**B**) The effects of resveratrol and pterostilbene on the invasion of HeLa adherent cells. The invasiveness of HeLa cells was analyzed using Matrigel-coated polycarbonate filters. The cells were incubated in the absence or presence of the two compounds (10 and 20 μM) for 48 h. The cells penetrating the filters were stained and counted using an optical microscope. * *p* < 0.05 versus the control.

To compare the effects of resveratrol and pterostilbene on the metastatic ability of cervical cancer cells, we examined whether the two compounds inhibit the migration and invasion of HeLa adherent cells. A monolayer wound healing assay was performed to evaluate their effects on cell migration. Pterostilbene more markedly decreased the migration of HeLa cells at both 24 and 48 h after treatment when compared to resveratrol (Figure 3A). The effects of the two compounds on cell invasion were assessed using a Matrigel-coated Transwell chamber system. Both resveratrol and pterostilbene resulted in a significant reduction in the invasiveness of HeLa cells (Figure 3B). In particular, the invasion inhibitory effect of pterostilbene was more potent than that of resveratrol. 

### 2.3. Comparison of the Cell Cycle Arrest and Apoptosis-Inducing Effects of Resveratrol and Pterostilbene in Cervical Cancer Cells

**Figure 4:**
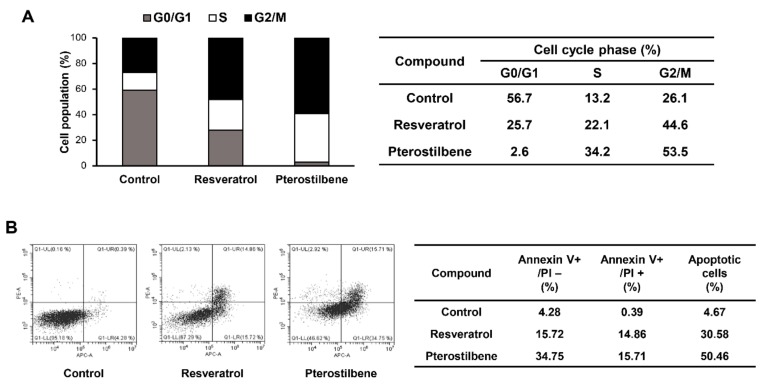
Effects of resveratrol and pterostilbene on the cell cycle and apoptotic cell death of HeLa cells. (**A**) The cell cycle distribution of HeLa adherent cells was evaluated by flow cytometry after the treatment of the two compounds (40 μM) for 48 h. (**B**) HeLa adherent cells were treated with resveratrol and pterostilbene (40 μM) for 48 h. Apoptotic cells were determined by flow cytometry analysis following annexin V-FITC and propidium iodide (PI) dual labeling.

**Figure 5:**
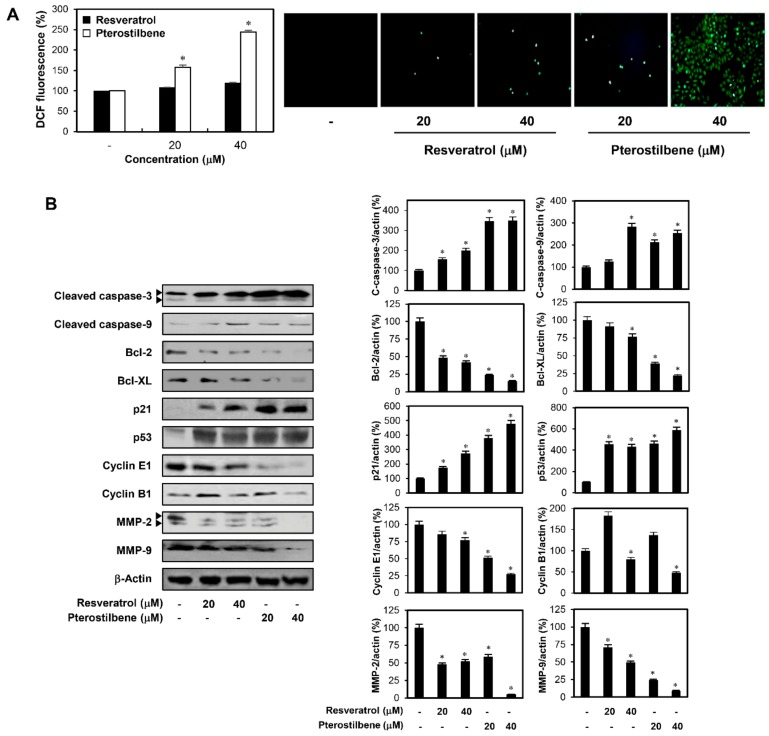
Identification of molecular mechanisms underlying the growth and migration inhibitory effects of resveratrol and pterostilbene in HeLa cells. (**A**) The effects of resveratrol and pterostilbene on reactive oxygen species (ROS) generation in HeLa adherent cells. The cells were treated with resveratrol and pterostilbene (20 and 40 μM) for 48 h. Intracellular ROS levels were detected with 2′,7′-dichlorofluorescein diacetate (DCFH-DA). (**B**) The effects of resveratrol and pterostilbene on the expression of cleaved caspase-3, cleaved caspase-9, Bcl-2, Bcl-XL, p21, p53, cyclin E1, cyclin B1, MMP-2, and MMP-9 in HeLa adherent cells. The cells were treated with the two compounds (20 and 40 μM) for 48 h, and the protein levels were detected by Western blot analysis using specific antibodies. The levels of β-actin were used as an internal control. Arrowheads indicate true bands for the molecular markers. * *p* < 0.05 versus the control.

To determine whether the growth inhibitory effects of resveratrol and pterostilbene on HeLa adherent cells were caused by cell cycle arrest, the effects of the two compounds on the cellular cell cycle distribution were quantified using flow cytometry analysis. Both resveratrol and pterostilbene induced cell cycle arrest at the S and G2/M phases along with a decrease in G0/G1 phase duration when compared with the control cells (Figure 4A). Notably, pterostilbene was more potent than resveratrol in blocking cell cycle progression. The induction of tumor suppressor protein p53 and its downstream target p21 can trigger cell cycle arrest by inhibiting the activity of cyclin-dependent kinase (CDK)–cyclin complexes [18]. Therefore, the effects of resveratrol and pterostilbene on the expression of these cell cycle regulators were assessed. Results revealed that the cell cycle arrest at the S and G2/M phases of HeLa adherent cells by resveratrol and pterostilbene was associated with the promotion of p53 and p21 expression and subsequent downregulation of cyclin E1 and cyclin B1 that are active in the S and G2 phases, respectively (Figure 5B). Furthermore, pterostilbene not only more significantly increased the expression levels of p53 and p21, but also decreased those of cyclin E1 and cyclin B1 compared to resveratrol. 

To further elucidate the mechanisms underlying the anticancer effects of the two compounds in cervical cancer cells, cellular apoptosis was quantitatively measured using flow cytometry analysis following annexinV-FITC/propidium iodide (PI) double staining. Annexin V is a marker of early apoptosis and PI is a marker of late apoptosis and necrosis. The total amount of early and late apoptotic cells was markedly increased after resveratrol and pterostilbene treatment in comparison with the control (Figure 4B). Moreover, the apoptosis-inducing effect of pterostilbene was stronger than that of resveratrol in HeLa adherent cells (from 4.67% to 30.58% and 50.46% by resveratrol and pterostilbene, respectively). The elevation of intracellular reactive oxygen species (ROS) plays an important role in mediating apoptotic processes [19]. Thus, to determine whether ROS are involved in the regulation of resveratrol- and pterostilbene-induced apoptosis, the levels of intracellular ROS in HeLa adherent cells were measured using the fluorescent 2′,7′-dichlorofluorescein diacetate (DCFH-DA) product. Pterostilbene more prominently elevated the production of ROS in comparison with resveratrol at the indicated doses (Figure 5A). In addition, the cell apoptosis induced by resveratrol and pterostilbene was involved in the activation of caspase-3 and caspase-9, as well as the downregulation of antiapoptotic proteins such as Bcl-2 and Bcl-XL (Figure 5B) [20]. These data also showed that pterostilbene was more effective in regulating the expression of these apoptosis-related proteins than resveratrol. 

Matrix metalloproteinases (MMPs) play a critical role in the degradation of the extracellular matrix (ECM) during cancer metastasis [21]. To further define the mechanism through which resveratrol and pterostilbene reduce cervical cancer cell migration and invasion, the protein expression levels of MMP-2 and MMP-9 in HeLa adherent cells were investigated. Pterostilbene more strongly suppressed the expression of MMP-2 and MMP-9 compared with resveratrol (Figure 5B). These findings suggest that pterostilbene may possess enhanced activity in inhibiting the metastasis of cervical cancer cells than resveratrol, through more effective downregulation of MMP-2 and MMP-9 expression.

Therefore, the superior growth and migration inhibitory effects of pterostilbene compared to resveratrol in HeLa adherent cells were mediated through the enhanced activation of multiple mechanisms, including cell cycle arrest at S and G2/M phases, induction of ROS-mediated caspase-dependent apoptosis, and inhibition of MMP-2/-9 expression.

### 2.4. Potent Inhibitory Activity of Pterostilbene against the Growth and Migration of Cervical CSCs

CSCs, which play critical roles in therapeutic resistance, recurrence, and metastasis of tumors, have been identified in various solid tumors and hematological cancers including cervical cancer [22,23]. Therefore, anticancer agents with the potential to eliminate cervical CSCs may provide novel therapeutic opportunities for more effective treatment of cervical carcinoma. 

**Figure 6:**
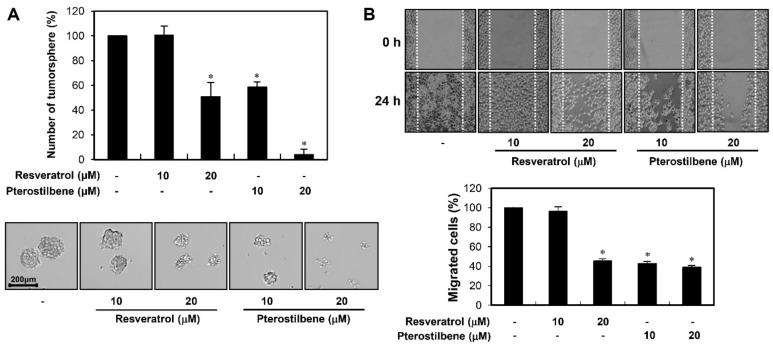
Effects of resveratrol and pterostilbene on the tumorsphere-forming ability and migration of cervical cancer stem cells (CSCs). (**A**) HeLa cancer stem-like cells were treated with the two compounds (10 and 20 μM) and incubated with the CSC culture media for eight days. The number of formed tumorspheres in each well was counted under a microscope. (**B**) HeLa cancer stem-like cells were seeded into laminin-coated culture plate and incubated with the CSC culture media in the absence or presence of resveratrol and pterostilbene (10 and 20 μM) for 24 h. The cells that migrated into the gap were counted under an optical microscope. White lines indicate the edge of the gap at 0 h. * *p* < 0.05 versus the control.

To assess the effects of resveratrol and pterostilbene against stem-like properties of cervical cancer cells, the CSC population from HeLa cells was enriched in serum-free suspended spheroid culture condition [24,25]. First, we examined whether the two compounds affect the clonogenic growth as tumorspheres of cancer stem-like cells derived from HeLa cells. The tumorsphere forming ability of HeLa cancer stem-like cells was significantly suppressed by treatment with resveratrol and pterostilbene (Figure 6A). They decreased both the number and size of HeLa cancer stem-like cells. Particularly, pterostilbene was much stronger in inhibiting the tumorsphere formation of HeLa cancer stem-like cells compared with resveratrol.

Next, we investigated the effects of resveratrol and pterostilbene on the migration of HeLa cancer stem-like cells. The wound healing assay showed that the two compounds reduced the migration of HeLa cancer stem-like cells when compared to the control conditions (Figure 6B). In addition, the migration inhibition activity of pterostilbene was much more potent than that of resveratrol. These findings underscore the superior therapeutic potential of pterostilbene to eliminate cervical CSCs.

### 2.5. Pterostilbene Exhibited Better Capacity for Inducing Cell Cycle Arrest and Apoptosis of Cervical CSCs Compared to Resveratrol

**Figure 7:**
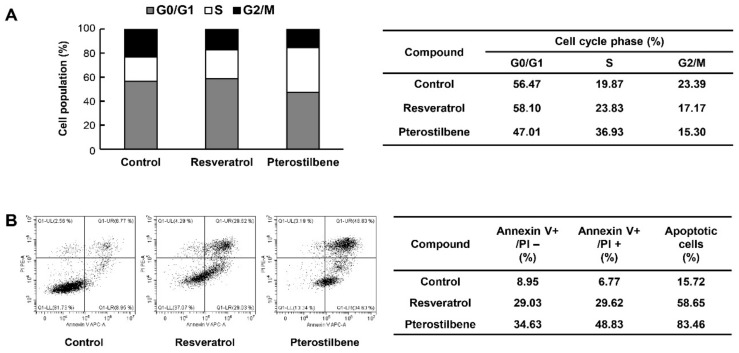
Effects of resveratrol and pterostilbene on the cell cycle and apoptotic cell death of cervical CSCs. (**A**) The cell cycle progression and (**B**) cellular apoptosis of HeLa cancer stem-like cells were measured by flow cytometry analysis after the treatment of resveratrol and pterostilbene (40 μM) for 48 h.

To further elucidate the inhibitory effects of resveratrol and pterostilbene on the growth of cervical CSCs, the cell cycle progression and cellular apoptosis of HeLa cancer stem-like cells were measured by flow cytometry analysis. As shown in Figure 7A, both resveratrol and pterostilbene induced S phase arrest (increase in the proportion of arrested cells from 19.87% to 23.83% and 36.93% by treatment with resveratrol and pterostilbene, respectively) when compared with the control cells. Moreover, pterostilbene more strongly induced cell cycle arrest than resveratrol in HeLa cancer stem-like cells. Our data also showed that the number of early and late apoptotic cells was markedly increased after resveratrol and pterostilbene treatment in comparison with the control (Figure 7B). The apoptosis promoting effect of pterostilbene was more potent compared to resveratrol in HeLa cancer stem-like cells (increase in the proportion of total apoptotic cells, from 15.72% to 58.65% and 83.46% by resveratrol and pterostilbene, respectively). These results indicate that pterostilbene has a better capacity for inducing cell cycle arrest and apoptosis of cervical CSCs than resveratrol, thereby causing a stronger inhibition in the tumorsphere-forming ability of cervical CSCs in comparison with resveratrol.

### 2.6. Effect of Pterostilbene on the Expression of Stemness Markers in Cervical CSCs 

**Figure 8:**
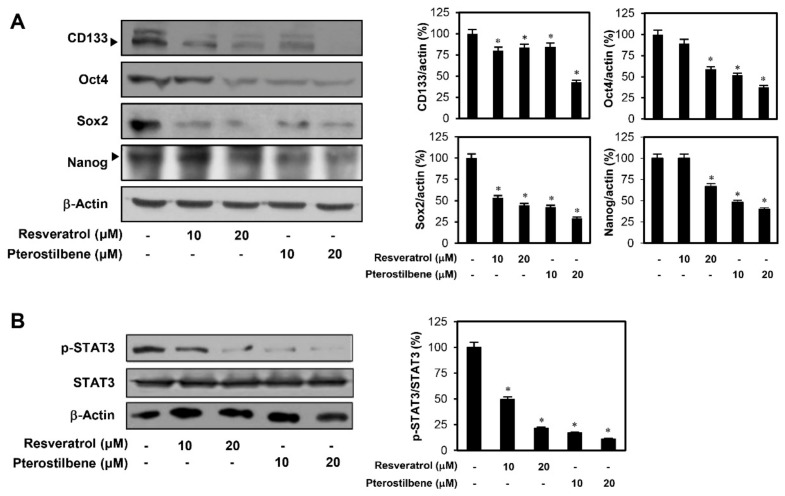
Effects of resveratrol and pterostilbene on stemness markers and signal transducer and activator of transcription 3 (STAT3) signaling in cervical CSCs. (**A**,**B**) HeLa cancer stem-like cells were treated with resveratrol and pterostilbene (10 and 20 μM) for 48 h, and the protein levels were detected by Western blot analysis using specific antibodies. The levels of β-actin were used as an internal control. Arrowheads indicate true bands for the molecular markers. * *p* < 0.05 versus the control.

To explore the mechanism by which resveratrol and pterostilbene inhibit the growth and migration of cervical CSCs, their effects on the expression of transcription factors, Sox2, Oct4, and Nanog, were investigated. These transcription factors have been reported to induce stem-like properties, in HeLa cancer stem-like cells [26,27,28]. The two compounds effectively reduced the expression levels of the key stemness-related transcription factors as well as CD133, a cell surface marker for CSCs, suggesting that the inhibitory effects of resveratrol and pterostilbene against cervical CSCs may be associated with the downregulation of these stemness regulators (Figure 8A). Pterostilbene treatment more noticeably decreased the expression levels of stemness markers compared with resveratrol treatment.

The STAT3 pathway is involved in the maintenance of cervical CSCs by regulating the expression of stem cell-related transcription factors [29,30]. To further understand the molecular mechanism underlying the anticancer effects of resveratrol and pterostilbene against cervical CSCs, we investigated whether the two compounds affect STAT3 signaling. Our results confirmed that the compounds significantly decreased the expression levels of phosphorylated STAT3, without inhibiting the total protein levels of STAT3 in HeLa cancer stem-like cells (Figure 8B). Furthermore, pterostilbene more profoundly inhibited the phosphorylation of STAT3 than resveratrol. These results demonstrate that pterostilbene is more effective in suppressing the stem-like properties of cervical cancer cells than resveratrol through stronger downregulation of specific CSC markers and STAT3 signaling.

## 3. Discussion

The health beneficial effects of stilbene, a class of natural polyphenolic compounds, have been extensively studied in the past several decades [10,11]. Resveratrol and pterostilbene, the most widely known stilbenes, have gained increasing attention due to their roles in the potential prevention of major non-infectious chronic diseases such as cancer, cardiovascular disease, diabetes, and neurological degeneration [12,13,14]. Although both resveratrol and pterostilbene possess the therapeutic activities to inhibit various mechanisms for these human diseases, the bioavailability of pterostilbene with two methoxy groups is higher than resveratrol with two hydroxyl groups [12]. According to several studies, resveratrol and pterostilbene exhibit bioavailability at approximately 20% and 80% in vivo, respectively [31,32]. Such structural differences between the two compounds are expected to make pterostilbene more easily absorbed by oral ingestion through the promotion of lipophilicity and membrane permeability, compared with resveratrol. 

The superior anticancer effects of pterostilbene have been reported in various tumors including lung, colon, breast, and cervical cancers [13]. Pterostilbene effectively suppressed cancer progression and metastasis by regulating apoptosis-dependent and apoptosis-independent signaling pathways. Accumulating evidence has shown that the anticancer effects of pterostilbene against cervical cancer are associated with the induction of apoptosis by activating the endoplasmic reticulum (ER)/nuclear factor erythroid 2-related factor 2 (Nrf2) pathway and downregulating the HPV oncoprotein E6 that causes the degradation of tumor suppressor protein p53 [17,33]. 

In the current study, we thoroughly investigated the cellular mechanisms responsible for the improved anticancer effects of pterostilbene compared to resveratrol in cervical cancer. Our results revealed that the superior growth and migration inhibitory effects of pterostilbene than resveratrol in HeLa cervical cancer cells could be attributed to the following reasons: the enhanced activation of multiple mechanisms, including cell cycle arrest at S and G2/M phases through the reduction of cyclin E1 and cyclin B1 expression following the induction of p53 and its downstream target p21; apoptosis through the activation of caspase-3 and caspase-9 mediated by ROS, as well as the downregulation of antiapoptotic proteins such as Bcl-2 and Bcl-XL; and the inhibition of MMP-2 and MMP-9 expression. 

In addition, this is the first study to investigate the suppressive activities of pterostilbene and resveratrol against cervical cancer stemness. The critical role of CSCs in cancer progression and metastasis has already been identified and validated in many studies [4,5,22,23]. Notably, CSCs display resistance to many types of therapies, which results in cancer recurrence. Thus, it is important to suppress the self-renewal, proliferation, and metastasis abilities of CSCs for more reliable cancer treatment. In several cancers, the therapeutic effects of pterostilbene to eradicate CSCs have been confirmed. Pterostilbene inhibits the tumorsphere formation, migration, and stemness-related gene expression of CD133+ CSCs by downregulating multifaceted oncoprotein (MUC1), NF-κB, and Wnt/β-catenin-dependent signaling pathways in lung, breast, brain, and liver tumors [34,35,36,37,38]. However, the inhibitory effect of pterostilbene against cervical CSCs has not been studied previously. 

In this study, pterostilbene significantly suppressed both the tumorsphere-forming ability and migration of HeLa cancer stem-like cells. Particularly, the therapeutic potential of pterostilbene to suppress cervical CSCs was markedly stronger than resveratrol. A tumorsphere is a solid, spherical structure developed from the proliferation of the cancer stem/progenitor cells. In these results, pterostilbene showed a better capacity for inducing cell cycle arrest and apoptosis of cervical CSCs in comparison with resveratrol. Therefore, the cell cycle arrest and apoptosis promoting effect of pterostilbene compared to resveratrol led to a stronger inhibition in the tumorsphere formation of HeLa cancer stem-like cells.

The stemness supporting transcription factors, such as Sox2, Klf4, c-Myc, Oct4, and Nanog, are upregulated in various types of CSCs [22,23,26,27,28]. Our results demonstrated that pterostilbene effectively decreased the expression levels of the major stemness transcription factors, including Sox2, Oct4, and Nanog, as well as a cell surface marker for CSCs, CD133, suggesting that the inhibitory effect of pterostilbene against cervical CSCs may be associated with the downregulation of these stemness regulators. In addition, pterostilbene more markedly suppressed the expression of stemness markers than resveratrol.

STAT3 is a transcription factor that is activated in many cancer types and can regulate pathways involving cell proliferation, cell survival, angiogenesis, and tumorigenesis [39]. Recent studies have revealed that STAT3 is an important regulator for self-renewal and survival of CSCs in various tumors [40]. In cervical carcinoma, STAT3 upregulates the stem-like characteristics of cervical cancer cells by increasing the expression of the stemness supporting markers such as Sox2, Oct4, and Nanog [29,30]. Therefore, targeting STAT3 signaling may be a promising approach to combat the survival of cervical CSCs. In the present study, pterostilbene resulted in a reduction in the expression levels of phosphorylated STAT3, without affecting the total protein levels of STAT3 in HeLa cancer stem-like cells. Furthermore, pterostilbene was more effective in suppressing the phosphorylation of STAT3 than resveratrol. 

Our results collectively demonstrate that pterostilbene can suppress the stem-like properties of cervical cancer cells by downregulating specific CSC markers and STAT3 signaling. Thus, pterostilbene might serve as a potential anticancer agent for more effectively eradicating cervical cancer by targeting both cancer cells and CSCs, with superior bioavailability compared to resveratrol. However, the precise mechanism underlying how pterostilbene modulates the phosphorylation of STAT3 remains unclear. It is well known that STAT3 is phosphorylated by various protein kinases, including epidermal growth factor receptor (EGFR), Janus kinases (JAK), and Src family kinases (SFKs) [41]. Accumulating evidence has revealed that pterostilbene and resveratrol can induce cell cycle arrest and apoptosis by inhibiting the upstream kinase activities of STAT3 signaling in several cancers such as breast, pancreatic, prostate, and bone tumors [42,43,44,45]. Further studies to understand the mechanism of action of pterostilbene against cervical CSCs will help the discovery of the upstream cellular mediators of STAT3 activity regulated by pterostilbene. Moreover, further in vivo experiments using tumor xenograft animal models will be required to better verify the therapeutic potential of pterostilbene for cervical cancer compared to resveratrol.

## 4. Materials and Methods 

### 4.1. Materials

Resveratrol and pterostilbene were purchased from Sigma-Aldrich (Saint Louis, MO, USA) and dissolved in dimethyl sulfoxide (DMSO) at a concentration of 100 mM to prepare a stock solution. Matrigel and gelatin were obtained from BD Biosciences (San Jose, CA, USA). Laminin and the Transwell chamber system were obtained from Koma Biotech (Seoul, Korea) and Corning Costar (Acton, MA, USA), respectively. Antibodies against p21, p53, MMP-2, MMP-9, Bcl-2, Bcl-XL, cleaved caspase-3, cleaved caspase-9, cyclin E1, cyclin B1, STAT3, phospho-STAT3, Sox2, Oct4, Nanog, β-actin, rabbit IgG, and mouse IgG were purchased from Cell Signaling Technology (Danvers, MA, USA). Anti-CD133 antibody was obtained from MiltenyiBiotec GmbH (BergischGladbach, Germany).

### 4.2. Cell Culture

Human cervical cancer HeLa, CaSki, and SiHa cell lines were obtained from the Korean Cell Line Bank (KCLB). The cells were cultured in Dulbecco’s modified Eagle medium (DMEM; Gibco, Grand Island, NY, USA) supplemented with 10% fetal bovine serum (FBS; Gibco) and 1% penicillin–streptomycin–amphotericin B (Lonza, Walkersville, MD, USA) and then maintained at 37 °C in a 5% CO_2_ humidified incubator.

### 4.3. Cell Growth Assay

HeLa, CaSki, and SiHa cells (3 × 10^3^ cells/well) were seeded in a 96-well culture plate and then treated with various concentrations of resveratrol and pterostilbene (0–200 μM) for 72 h. Cell growth was measured with the 3-(4,5-dimethylthiazol-2-yl)-2,5-diphenyltetrazolium bromide (MTT) colorimetric assay (Sigma-Aldrich). The absorbance of each well was determined at a wavelength of 540 nm using a microplate reader (Thermo Fisher Scientific, Vantaa, Finland). The IC_50_ values from obtained data were analyzed using the curve-fitting program GraphPad Prism 5 (GraphPad Software, La Jolla, CA, USA).

### 4.4. Colony Formation Assay

To evaluate the colony forming inhibitory effects of resveratrol and pterostilbene, HeLa, CaSki, and SiHa cells (2.5 × 10^2^ cells/well) were seeded in a six-well culture plate. After 24 h incubation, the cells were treated with resveratrol and pterostilbene (10 and 20 μM) and incubated for 7 days until colonies were formed. Following this, the colonies were fixed with 4% formaldehyde and stained with 0.5% crystal violet solution for 10 min and washed with double-distilled water. The number of colonies on each well was counted and the percentage of compound-treated colonies relative to DMSO-treated control colonies was calculated.

### 4.5. Wound Healing Assay

HeLa cells (2.5 × 10^5^ cells/well) were seeded in a 24-well culture plate. The confluent monolayer cells were scratched using a tip and each well was washed with PBS to remove non-adherent cells. The cells were treated with resveratrol and pterostilbene (20 μM) and then incubated for up to 48 h. The perimeter of the central cell-free zone was observed under an optical microscope (Olympus, Center Valley, PA, USA).

### 4.6. Invasion Assay

The invasiveness of cells was examined using Transwell chamber inserts with a pore size of 8.0 μm. The lower surface of the polycarbonate filter was coated with 10 μL of gelatin (1 mg/mL), while the upper surface was coated with 10 μL of Matrigel (3 mg/mL). HeLa cells (5 × 10^4^ cells/well) were seeded in the upper chamber of the filter; resveratrol and pterostilbene (10 and 20 μM) were added to the lower chamber filled with medium. The chamber was incubated at 37 °C for 48 h, and then the cells were fixed with 70% methanol and stained with hematoxylin and eosin (H and E). The total number of cells that invaded the lower chamber of the filter was counted using an optical microscope (Olympus).

### 4.7. Cell Cycle Analysis

The cell cycle distribution was analyzed using the Muse Cell Cycle Assay Kit (Millipore, Hayward, CA, USA) according to the manufacturer’s instructions. To this end, HeLa cells (5 × 10^5^ cells/dish) were seeded in a 60-mm culture dish and treated with resveratrol and pterostilbene for 48 h. The cells were collected by centrifugation and washed using PBS and fixed in ice cold 70% ethanol at −20 °C for more than 3 h. The cells were then incubated with the Muse Cell Cycle reagent for 30 min at room temperature in the dark. The cell cycle analysis was carried out using the Muse Cell Analyzer (Millipore).

### 4.8. Apoptosis Analysis

The apoptotic cell distribution was determined using the Muse Annexin V and Dead Cell Kit (Millipore) according to the manufacturer’s instructions. Briefly, after treatment with resveratrol and pterostilbene, HeLa cells were collected and diluted with PBS containing 1% bovine serum albumin (BSA) as a dilution buffer to a concentration of 5 × 10^5^ cells/mL. The single cell suspension was mixed with the Muse Annexin V/Dead Cell reagent at a 1:1 ratio and incubated in the dark for 20 min at room temperature. The cells were then analyzed using the Muse Cell Analyzer (Millipore).

### 4.9. Reactive Oxygen Species (ROS) Measurement

Intracellular ROS levels were measured using a ROS-sensitive fluorescence indicator, 2′,7′-dichlorofluorescein diacetate (DCFH-DA; Sigma-Aldrich). HeLa cells (1 × 10^5^ cells/well) were seeded in a 96-black well culture plate and treated with resveratrol and pterostilbene (20 and 40 μM) for 48 h. The cells were then incubated with 10 μM of DCFH-DA for 20 min and washed with PBS. The fluorescence intensity of DCF was detected using a multimode microplate reader (Biotek, Inc., Winooski, VT, USA) at the excitation and emission wavelengths of 495 and 529 nm, respectively. The fluorescent images were also acquired using an Optinity KI-2000F fluorescence microscope (Korea Lab Tech, Seong Nam, Korea).

### 4.10. Western Blot Analysis

Cells were lysed using RIPA buffer (Sigma-Aldrich) supplemented with a protease inhibitor cocktail (Roche Diagnostics, Mannheim, Germany) on ice. Protein concentrations of the extracts were determined using a BCA Protein Assay kit (Pierce; Thermo Fisher Scientific, Inc., Waltham, MA, USA). Equal amounts of cell lysate were separated by 10% sodium dodecyl sulfate-polyacrylamide gel electrophoresis (SDS-PAGE), and the separated proteins were transferred to polyvinylidene difluoride (PVDF) membranes (EMD Millipore) using standard electroblotting procedures. The blots were blocked in Tris-buffered saline with Tween-20 (TBST) containing 5% skim milk at room temperature for 1 h and immunolabeled with primary antibodies against p21, p53, MMP-2, MMP-9, Bcl-2, Bcl-XL, cleaved caspase-3, cleaved caspase-9, cyclin E1, cyclin B1, STAT3, phospho-STAT3, Sox2, Oct4, Nanog, CD133 (dilution 1:2000), and β-actin (dilution 1:10000) overnight at 4 °C. After washing with TBST three times, the membranes were incubated with horseradish peroxidase-conjugated anti-rabbit or anti-mouse (dilution 1:3000) secondary antibody for 1 h at room temperature. Immunolabeling was detected with an enhanced chemiluminescence (ECL) kit (Bio-Rad Laboratories, Inc., Hercules, CA, USA) according to the manufacturer’s instructions. The band density was analyzed using ImageJ software (version 1.5; NIH).

### 4.11. CSC Culture

CSCs were cultured using a non-adhesive culture method [24,25]. To propagate cervical cancer stem-like cells, HeLa cells grown in the serum-based media were cultured in Dulbecco’s modified Eagle medium/nutrient mixture F-12 (DMEM/F12; Gibco) containing 1× B-27 serum-free supplement (Gibco), 5 μg/mL heparin (Sigma-Aldrich), 2 mM L-glutamine (Gibco), 20 ng/mL epidermal growth factor (EGF; Gibco), 20 ng/mL basic fibroblast growth factor (bFGF; Gibco), and 1% penicillin/streptomycin (Gibco). Tumorspheres grown in the serum-free media were subcultured every 7 days by dissociating with Accutase (Millipore) and maintained at 37 °C in a 5% CO_2_ humidified incubator.

### 4.12. CSC Tumorsphere Formation Assay

HeLa cancer stem-like cells were seeded in a 96-well culture plate at a density of 500 cells/well using the serum-free media with EGF and bFGF. After 8 days of resveratrol and pterostilbene treatment (10 and 20 μM), the number of tumorspheres formed in each well was counted under an optical microscope (Olympus).

### 4.13. CSC Migration Assay

For the CSC migration assay, the ibidi culture inserts (IBIDI GmbH, Martinsried, Germany) were placed in a laminin-coated 24-well culture plate. HeLa cancer stem-like cells were prepared at a density of 5 × 10^5^ cells/mL, of which 70 μL was transferred to each chamber. After cell attachment for 24 h, the culture inserts were removed, and the attached cells were incubated with the serum-free media containing EGF and bFGF, in the absence or presence of resveratrol and pterostilbene (10 and 20 μM) for 24 h. The perimeter of the central cell-free zone was confirmed under an optical microscope (Olympus).

### 4.14. Statistical Analysis

The data were presented as the mean ± standard error (SE) of three independent experiments. Student’s *t*-test was used to determine statistical significance between the control and the test groups. A *p*-value of <0.05 was considered to indicate a statistically significant difference.

## 5. Conclusions

The present study focused on the therapeutic effect and mechanism underlying the anticancer effects of pterostilbene against both cancer cells and cancer stem-like cells in cervical cancer compared to resveratrol. Our results demonstrated that the superior inhibitory effects of pterostilbene compared to resveratrol were associated with the enhanced activation of multiple mechanisms, including cell cycle arrest at S and G2/M phases through the induction of p53 and p21 and subsequent reduction of cyclin E1 and cyclin B1; apoptosis through the activation of caspase-3 and -9 mediated by ROS and the downregulation of Bcl-2 and Bcl-XL antiapoptotic proteins; and the inhibition of MMP-2 and -9 expression. Notably, pterostilbene exhibited a greater inhibitory effect against HeLa cancer stem-like cells than resveratrol through more potent inhibition of the expression levels of stemness markers, such as CD133, Oct4, Sox2, and Nanog, as well as STAT3 signaling. Based on these findings, we conclude that pterostilbene is a better potential candidate than resveratrol for more effectively treating cervical carcinoma.
